# Heterozygous *Nme7* Mutation Affects Glucose Tolerance in Male Rats

**DOI:** 10.3390/genes12071087

**Published:** 2021-07-18

**Authors:** Lucie Šedová, Jan Prochazka, Dagmar Zudová, Běla Bendlová, Josef Včelák, Radislav Sedlacek, Ondřej Šeda

**Affiliations:** 1 Laboratory of Transgenic Models of Diseases, Division BIOCEV, Institute of Molecular Genetics of the Czech Academy of Sciences, 252 50 Vestec, Czech Republic; radislav.sedlacek@img.cas.cz; 2 Institute of Biology and Medical Genetics, First Faculty of Medicine, Charles University, and General University Hospital, 128 00 Prague, Czech Republic; oseda@lf1.cuni.cz; 3 Czech Centre for Phenogenomics, Institute of Molecular Genetics of the Czech Academy of Sciences, v.v.i., 252 50 Vestec, Czech Republic; jan.prochazka@img.cas.cz (J.P.); dagmar.zudova@img.cas.cz (D.Z.); 4 Department of Molecular Endocrinology, Institute of Endocrinology, 116 94 Prague, Czech Republic; bbendlova@endo.cz (B.B.); JVcelak@endo.cz (J.V.)

**Keywords:** metabolic syndrome, pancreatic fibrosis, animal models

## Abstract

Complex metabolic conditions such as type 2 diabetes and obesity result from the interaction of numerous genetic and environmental factors. While the family of Nme proteins has been connected so far mostly to development, proliferation, or ciliary functions, several lines of evidence from human and experimental studies point to the potential involvement of one of its members, NME7 (non-metastatic cells 7, nucleoside diphosphate kinase 7) in carbohydrate and lipid metabolism. As a complete lack of *Nme7* is semilethal in rats, we compared morphometric, metabolic, and transcriptomic profiles of standard diet-fed heterozygous *Nme7+/*− on male rats vs. their wild-type *Nme7+/+* controls. *Nme7+/*− animals showed increased body weight, adiposity, higher insulin levels together with decreased glucose tolerance. Moreover, they displayed pancreatic islet fibrosis and kidney tubular damage. Despite no signs of overt liver steatosis or dyslipidemia, we found significant changes in the hepatic transcriptome of *Nme7+/*− male rats with a concerted increase of expression of lipogenic enzymes including *Scd1*, *Fads1*, *Dhcr7* and a decrease of *Cyp7b1* and *Nme7*. Network analyses suggested possible links between *Nme7* and the activation of Srebf1 and Srebf2 upstream regulators. These results further support the implication of NME7 in the pathogenesis of glucose intolerance and adiposity.

## 1. Introduction

Diabetes and obesity partake in a combined epidemic of “diabesity”, affecting hundreds of millions of people worldwide. Both diabetes and obesity increase the risk for cardiovascular disease and several types of cancer, boosting the associated morbidity and mortality, which translates into the soaring expenditure of public health systems. The conditions arise from the complex and dynamic interplay between genetic and environmental factors [1,2]. Many of the molecular mechanisms and underlying gene networks are shared within diabesity [3]. Furthermore, it is not uncommon that risk genetic variants affect multiple pathways and conditions. The genome-wide pleiotropy was reported in large studies, e.g., for body mass index and coronary artery disease [4], diabetes and coronary heart disease [5], or diabetes and obesity [6]. In the process of establishing the contribution of a particular gene to such a multifactorial condition, genetically designed animal models represent an invaluable tool [7]. Following the initial pilot observation of an association of variants of the NME7 (nucleoside diphosphate kinase 7, non-metastatic cells 7) gene to indices of diabetes and dyslipidemia in two unrelated human populations [8], we analyzed the *Nme7* interactome in a panel of recombinant inbred rat strains combining alleles of two inbred models of metabolic syndrome [9]. The rat strains with the lowest hepatic expression of *Nme7* had the most profound perturbations in carbohydrate and lipid metabolism networks [10]. Therefore, we generated the *Nme7* k.o. rat model to assess the role of *Nme7* in aspects of carbohydrate and lipid metabolism. NME7 is a recognized member of ciliome [11,12] and has a regulatory role in microtubule nucleating activity of γ-tubulin ring complex, the primary microtubule nucleator in animal cells [13]. The phenotypes arising from NME7 deficiency range from situs inversus was reported in two siblings from a consanguineous family [14], through situs inversus, hydrocephalus and excessive nasal exsudates in mice [15,16] to situs inversus, sterility and massive hydrocephalus leading to premature death in rats [17]. As the systemic *Nme7* deficiency was semilethal, we proceeded to compare the morphometric, metabolic, and transcriptomic profiles of heterozygous *Nme7+/*− rats vs. their wild-type *Nme7+/+* controls.

## 2. Materials and Methods 

### 2.1. Animals

All animal studies were ethically reviewed and performed in accordance with European directive 2010/63/EU and were approved by the Czech Central Commission for Animal Welfare. The Sprague Dawley (SD) rats were acquired in-house from the rodent colony kept at the Czech Centre for Phenogenomics, Prague (https://www.phenogenomics.cz/). Rats were housed and handled according to the institutional committee guidelines with free access to food and water. From weaning, two rats per cage were housed together in the individually-ventilated cages till the end of the experiment in the controlled SPF (specific pathogen-free) environment: 12h light/dark cycles (6:00 a.m./6:00 p.m.), humidity 45–60%, temperature 20–22 °C.

### 2.2. Genotyping

As described previously [17], the *Nme7*-knockout rat was generated using CRISPR/Cas9 nuclease system by targeting the exon 4 of the *Nme7* gene. This led to a 5-nucleotide deletion, resulting in the creation of a premature stop codon. In the process of the original derivation of the SD*^Nme7^* line, we established a genotyping protocol, subjecting the isolated genomic DNA to PCR using primers Nme7R: ‘5′-CCACAGTTAGATGAGGACTAGG-3’ and Nme7F: ‘5′-TGTGTGTCACCACACCCAGC-3’. The PCR product was subsequently cut using TaqI restriction enzyme (Thermo Fisher Scientific, Waltham, MA, USA). Due to the introduced 5-nt (TCGAA) deletion, one TaqI cleavage site was lost; therefore, only 2 fragments were detected in DNA carrying the 5-nt deletion instead of the 3 fragments present in SD wild type rats, as described previously [17].

### 2.3. Experimental Protocol

All rats were weaned at the age of 4 weeks. Throughout the experiment, the rats were fed standard chow. Body weight was measured weekly, starting at weaning, both in SD male rats (wild-type, wt hereafter, *n* = 10) and in the heterozygous SD*^Nme7+/−^* male rats (*Nme7+/−* hereafter, *n* = 17). At the age of 12 weeks, an intraperitoneal glucose tolerance test was performed. At the age of 14 weeks, rats were trained for 1-day acclimation in metabolic cages. One week later, indirect calorimetry measurement was performed using TSE Phenomaster system. At the age of 17 weeks, an oral glucose tolerance test was performed. Finally, rats were sacrificed after overnight fasting at the age of 20–21 weeks, blood and organ samples were collected for further analyses.

#### 2.3.1. Glucose Tolerance Tests

Intraperitoneal glucose tolerance test (IPGTT) was performed after 16-h fasting according to standard operating procedures of Czech Centre for Phenogenomics. In short, 20% glucose solution was used for intraperitoneal injection, and the volume of the injection was calculated so that each rat received 2 g of glucose per 1 kg of body weight. Blood glucose was analyzed before intraperitoneal injection and at 15, 30, 60, and 120 min after injection. In addition, areas under the glycemic curves were calculated. Oral glucose tolerance test was performed after 16-h fast using intragastric glucose administration to conscious rats by oral gavage (3 g/kg body weight, 30% aqueous solution); blood glucose was analyzed before and at 15, 30, 60, 120, and 180 min after bolus administration using Ascensia EliteBlood Glucose Meter (Bayer HealthCare, Mishawaka, IN, USA).

#### 2.3.2. Biochemistry

All analytes except for insulin were measured by the Beckman Coulter AU480 using Li-heparinized plasma at sacrifice according to standard operating procedures of the Czech Centre for Phenogenomics. Insulin levels were measured by Ultrasensitive Rat Insulin ELISA (10-1251-01) kit, Mercodia (Winston Salem, NC, USA).

#### 2.3.3. Indirect Calorimetry

Indirect calorimetry raw data were acquired using a standardized TSE Phenomaster protocol of the Czech Centre for Phenogenomics. Shortly after, one week before the measurement, rats were trained for a 1-day acclimation stay in metabolic cages. For 24 h, oxygen and carbon dioxide concentrations, temperature, air flow, activity, food and water intake were measured in 7 *Nme7+/−* and 6 wt male rats. From these parameters, oxygen consumption (mL/kg/h), carbon dioxide production (mL/kg/h), respiratory exchange rate (VCO_2_/VO_2_) and heat production were calculated (kcal/(kg·h)). Data are presented according to 12 h light/dark cycles.

### 2.4. Gene Expression

Total RNA was isolated from liver tissue and epididymal (visceral) fat in male rats (RNeasy Mini Kit, Qiagen; Hilden, Germany). Total RNA was assessed for quality and integrity using Agilent 2100 Bioanalyzer system (Agilent, Palo Alto; Santa Clara, CA, USA). Microarray experiments were performed using the Rat Gene 2.1 ST Array Strip. The hybridization procedure was completed using the Affymetrix GeneAtlas^®^ system according to the manufacturer’s instructions, including recommended quality filters. Transcriptomic data were normalized using Robust Multichip Average (RMA) algorithm, the set of obtained differentially expressed probesets was subsequently filtered by a false discovery rate (FDR) method implemented in PARTEK Genomics Suite 7 (Partek, St. Louis, MO, USA). Transcriptomic data were then processed by a standardized sequence of analyses including gene ontology, ‘Mechanistic Networks’ and ‘Causal Network Analysis’ using Ingenuity Pathway Analysis. The microarray data generated during and/or analyzed during the current study are available in the ArrayExpress repository (https://www.ebi.ac.uk/arrayexpress), Experiment ArrayExpress accession: E-MTAB-10011. To validate microarray gene expression data, we performed quantitative real-time PCR (RT-qPCR) using TaqMan^®^ probes (Applied Biosystems^TM^) according to the manufacturer’s instructions. Total RNA (1 µg) was reverse-transcribed with oligo-dT primers using the SuperScript IV (Invitrogen). Real-time PCR reaction was performed in triplicate with TaqMan^®^ Gene Expression Master Mix (Applied Biosystems) according to the manufacturer’s protocol (Invitrogen) using Applied Biosystems^®^ 7900HT Real-Time PCR System. Results were analyzed using the Livak analysis method [18] with glyceraldehyde 3-phosphate dehydrogenase (*Gapdh*) as the reference gene. The probes used for validation were Rn00593500_m1 for *Nme7*, Rn00584915_m1 for fatty acid desaturase (*Fads1*), Rn01461862_m1 for cytochrome P450, family 7, subfamily b, polypeptide 1 (*Cyp7b1*), Rn00574380_m1 for insulin-induced gene 1 (*Insig1*), Rn00574366_m1 for 7-dehydrocholesterol reductase (*Dhcr7*), Rn00594894_g1 for stearoyl-Coenzyme A desaturase 1 (*Scd1*), Rn00589521_m1 for suppressor of cytokine signaling 2 (*Socs2*), Rn00577779_m1 for hydroxysteroid 17-β dehydrogenase 2 (*Hsd17b2*), and Rn00710306_m1 for insulin-like growth factor 1 (*Igf1*).

### 2.5. Histology

Sampled organs were fixed for 24 h in a 10% formalin solution and processed by standard histological methods using an automated tissue processor (Leica ASP6025, Leica Microsystems, Wetzlar, Germany) and then embedded in paraffin blocks using a Leica EG 1150H paraffin embedding station (Leica Microsystems, Wetzlar, Germany). Three to five μm-thick slices were cut from each sample using a microtome (Leica RM2255, Leica Microsystems, Wetzlar, Germany) and mounted on standard glass slides (Bammed, Czech Republic). The slices were stained with hematoxylin-eosin (DiaPath, Martinengo, Italy) using Ventana Symphony H&E Slide Stainer (Ventana Medical Systems, Inc., Oro Valley, AZ, USA), islet fibrosis was manifested using Masson trichrome staining. The prepared samples were evaluated as light microscopic images obtained using a Carl Zeiss Axio Scope A1 (Zeiss, Oberkochen, Germany) and the Axio Scan.Z1 slide scanner (Zeiss, Oberkochen, Germany). Immunohistochemistry was performed using commercially available antibodies (Abcam, Cambridge, MA, USA) against *Nme7* (ab220753), insulin (ab7842), glucagon (ab167078), secondary antibodies HRP Goat-anti-rabbit (ab205718) and Goat-anti-guinea pig (ab7140) according to manufacturer’s instructions. The sizes of Langerhans islets and adipocytes was measured in 6 *Nme7+/–* and 5 wt male rats using Zen 3.2 (Zeiss, Oberkochen, Germany). In every animal, we measured the length and the width of 25 Langerhans islets. The adipocyte area was measured in 50 adipocytes per animal. Altogether, the pancreas was histologically examined in 18 male rats (13 *Nme7+/−* and 5 wt), liver in 14 male rats (9 *Nme7+/−* and 5 wt), the epididymal fat pad in 12 male rats (7 *Nme7+/−* and 5 wt) and kidney in 12 male rats (7 *Nme7+/−* and 5 wt).

### 2.6. Statistical Analysis

All statistical analyses were performed using STATISTICA 14 (Tibco, München, Germany). When comparing morphometric and biochemical variables between two groups, the statistical analysis was performed by unpaired (two-tailed) Student *t* test. Repeated measures ANOVA test was used for weight timecourse, IPGTT and OGTT. Two-way ANOVA with post hoc Tukey multiple comparisons test was used for indirect calorimetry measurements. Benjamini–Hochberg procedure was applied to control the false discovery rate (FDR) [19]. *p* values < 0.05 were considered significant. In transcriptome assessment, the correction for multiple comparisons was performed by applying the FDR < 0.05 in PARTEK Genomics Suite, followed by filtering out transcripts with an expression difference less or equal to 1.2-fold in the particular tissue between SD*^Nme7+/^^–^* and SD rats. Only transcripts passing these criteria were then subjected to analyses in Ingenuity Pathways Analysis described above, where Benjamini–Hochberg multiple testing correction was applied for Upstream Regulator, Causal Network, Canonical Pathway, Disease or Function analyses [20].

## 3. Results

### 3.1. Increased Body Weight and Adiposity in Nme7+/− Rats

**Figure 1:**
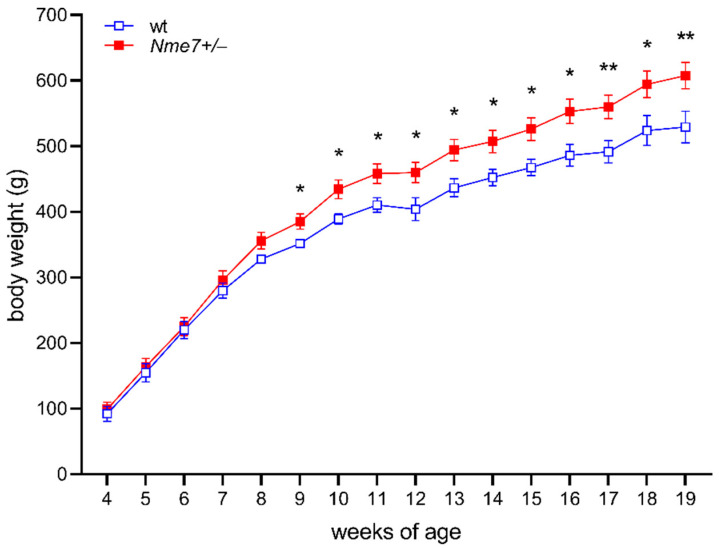
Bodyweight time course of wild-type (wt, open blue squares, *n* = 10) and *Nme7+/−* heterozygous (*Nme7+/−*, red closed squares, *n* = 17) male rats: Data are expressed as mean ± SEM; the significance levels for strain comparison using the repeated measures ANOVA are indicated as follows: * *p* < 0.05, ** *p* < 0.01 for differences between *Nme7+/−* and wt rats.

In the first eight weeks, the body weights did not differ between *Nme7+/*− and wild-type rats. However, *Nme7+/*− males were heavier from the week 9 onwards compared to their wild-type controls (Figure 1). The weights of liver, heart, kidney, spleen, and brown fat per 100 g of body weight assessed at the end of the experiment did not show any genotype-related differences. The weight of epididymal fat per 100g of bodyweight in *Nme7+/*− rats was slightly higher in comparison with wt controls (*Nme7+/*−: 1.61 ± 0.13 vs. wt: 1.22 ± 0.11 g/100 g bodyweight, *p* = 0.037).

### 3.2. Impaired Glucose Tolerance of Nme7+/− Rats

**Figure 2:**
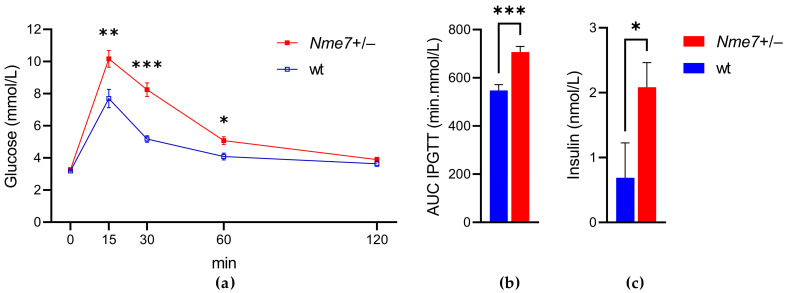
The intraperitoneal glucose tolerance test (IPGTT): (**a**) The course of glycaemic curves in wild-type (wt, open blue squares, *n* = 10) and *Nme7+/*− heterozygous (*Nme7+/*−, red closed squares, *n* = 17) male) rats. (**b**) Areas under the glycaemic curves (AUC) and (**c**) fasting insulin in wild-type (wt, blue bars) and *Nme7+/*− heterozygous (*Nme7+/*−, red bars) rats. Data are expressed as mean ± SEM; the significance levels for strain comparison using the repeated measures ANOVA (IPGTT) or the unpaired (two-tailed) Student t-test (AUC, insulin) are indicated as follows: * *p* < 0.05, ** *p* < 0.01, *** *p* < 0.001.

The *Nme7+/−* rats showed impaired glucose tolerance compared to their respective wild-type controls. The elevation of glycemia was evident in the initial 30 min of intraperitoneal glucose tolerance test (Figure 2a), resulting in significantly higher areas under the glycemic curves in *Nme7+/−* rats (Figure 2b). Furthermore, *Nme7+/−* rats showed a higher concentration of fasting insulin than wild-type animals (*p* = 0.029; Figure 2c). The impaired glucose tolerance was confirmed in *Nme7+/−* rats by the oral glucose tolerance test 5 weeks later (at the age of 17 weeks, Supplementary Figure S1).

### 3.3. Metabolic and Biochemical Profile of Nme7+/− and wild-Type Rats

**Figure 3:**
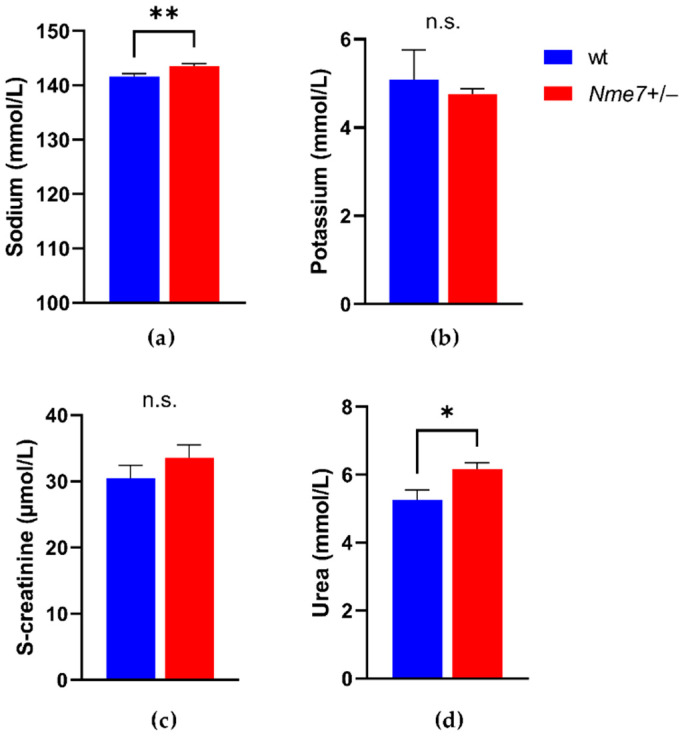
(**a**) Plasma sodium; (**b**) potassium; (**c**) creatinine; (**d**) urea in wild-type (wt, blue, *n* = 10) and *Nme7+/*− heterozygous (*Nme7+/*−, red, *n* = 17) male rats. Data are expressed as mean ± SEM; the significance levels for strain comparison using the unpaired (two-tailed) Student t-test are indicated as follows: n.s. not significant, * *p* < 0.05, ** *p* < 0.01.

**Figure 4:**
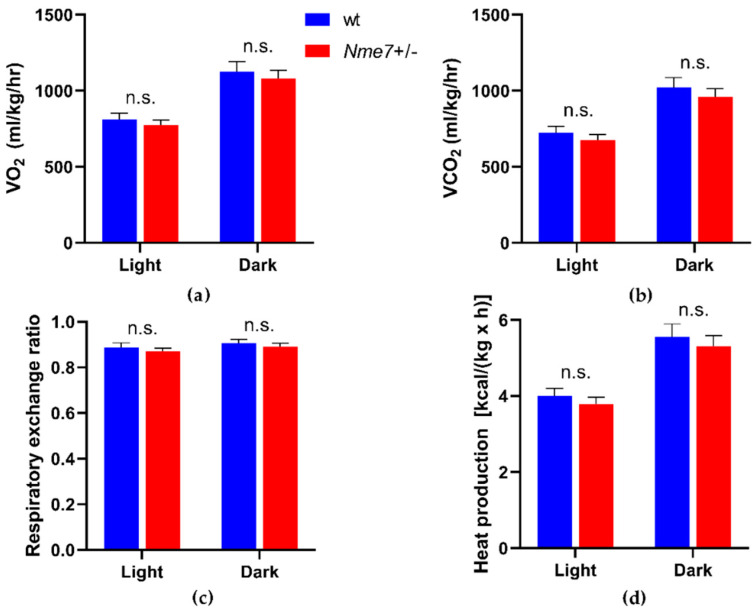
(**a**) Quantification of O_2_ consumption; (**b**) Quantification of carbon dioxide release; (**c**) Respiratory exchange ratio; (**d**) Heat production in wild-type (wt, blue, *n* = 6) and *Nme7+/*− heterozygous (*Nme7+/*−, red, *n* = 7) male rats, corrected for bodyweight. Data are expressed as mean ± SEM; the strain comparison using the post-hoc Fisher’s least significant difference test of the two-way ANOVA for STRAIN and PERIOD as major factors are indicated in the graph as follows: n.s. not significant.

We did not observe any genotype differences in concentrations of triacylglycerols, total and HDL cholesterol, alkaline phosphatase, aspartate aminotransferase, total protein, bilirubin, phosphate, chloride (Supplementary Figure S2), potassium, and creatinine (Figure 3). *Nme7+/*− males showed higher level of plasma sodium (*p* = 0.009), urea (*p* = 0.008) (Figure 3), and calcium (*p* = 0.007) compared to wild-type males. The plasma iron concentration was lower in *Nme7+/*− than in wt animals (*p* = 0.002) (Supplementary Figure S2). Haematologic parameters of the two groups were comparable except for a slightly higher count of white blood cells and lymphocytes in *Nme7+/*− (Supplementary Table S1). There were no differences between *Nme7+/−* and wt in any of the main measures of energy expenditure when corrected for bodyweight as measured by the indirect calorimetry (Figure 4).

### 3.4. Histological Assessment of Nme7+/− and Wild-Type Male Rats

**Figure 5:**
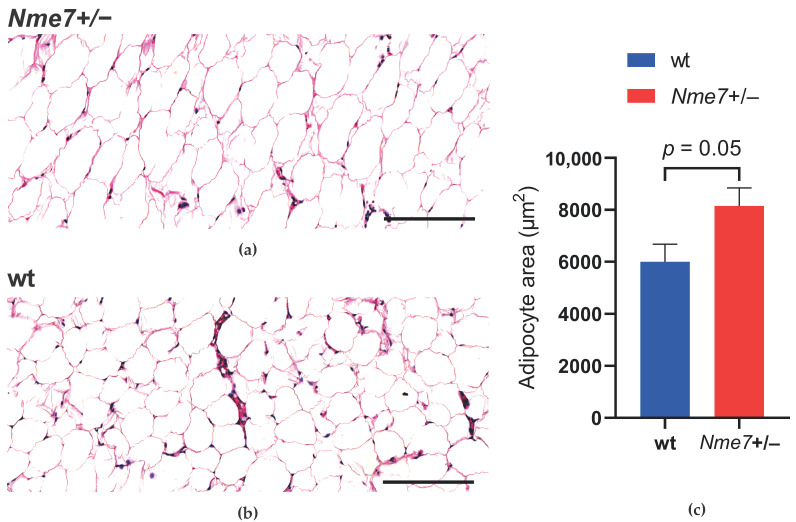
Adipose tissue of epididymal fat pad in *Nme7+/*− heterozygous (**a**) and wild-type (**b**) rats, hematoxylin-eosin staining, bars indicate 200 μm; (**c**) Adipocyte area (epididymal fat pad) of wild-type (wt, blue, *n* = 5) and *Nme7+/*− heterozygous (*Nme7+/*−, red, *n* = 6) male rats. Data are expressed as mean ± SEM; the significance level for strain comparison using the unpaired (two-tailed) Student t-test is indicated in the graph.

**Figure 6:**
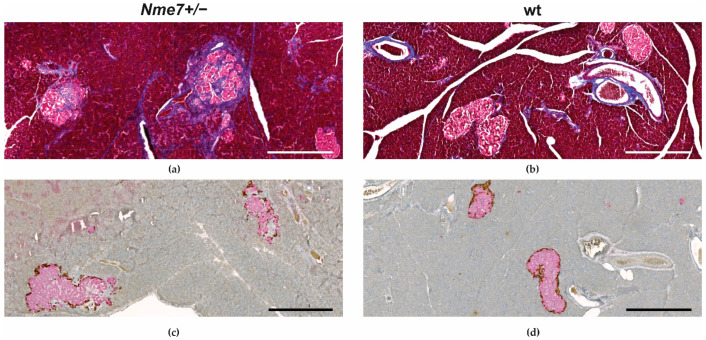
Representative islet changes in *Nme7 +/*– male rats: Irregular-border islets with fibrotic collagenous tissue segregating the functional areas of β-cells (**a**) compared to non-fibrotic islets of wt male controls. Masson trichrome stains the collagen fibers in blue (**a**,**b**). Insulin (pink) and glucagon (brown) staining of pancreas show enlarged and disrupted islets in *Nme7+/*− male rats (**c**) compared to compact islets in wt male rats (**d**). Bars indicate 500 μm.

We have histologically examined the liver, white adipose tissue, pancreas, and kidney. There were no apparent morphological differences in liver tissue between *Nme7+/−* and wt rats (Supplementary Figure S3). The assessment of white adipose tissue within the epididymal fat pads revealed that the mean adipocyte size in *Nme7+/−* was bigger compared to wt controls (Figure 5). There was no statistical difference between *Nme7+/−* male rats and SD male control rats in pancreatic islet size. Only *Nme7+/−* male rats displayed fibrotic islets, disrupted by bands of collagenous tissue (Figure 6a,b). Insulin-glucagon staining revealed that functional parts of islets were separated by fibrous tissue (Figure 6c,d). In *Nme7+/−* kidneys, we observed hemorrhage and tubular damage, such as detached necrotic tubular epithelial cell lining and protein cast formation in tubules (Supplementary Figure S3). We did not find any differences in glomeruli number and size (data not shown).

### 3.5. Expression of Nme7 and Transcriptome Profile of Nme7+/− and Wild-Type Rats

**Figure 7:**
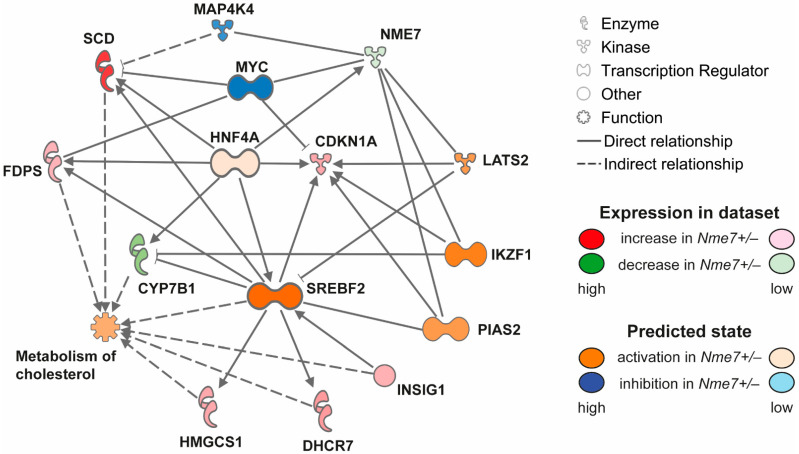
Mechanistic network reaching the highest score for comparison of *Nme7+/*− vs. wild-type hepatic transcriptomes, showing effects on activation (shades of orange) or inhibition (shades of blue) of upstream regulators. Transcripts significantly differentially expressed are displayed in shades of green (downregulation) or red (upregulation).

We assessed the expression of *Nme7* in the liver, kidney, white adipose (epididymal) tissue, and pancreas of *Nme7+/−* male rats. In all tested tissues, *Nme7+/−* animals showed lower expression of *Nme7*. The comparison of white adipose tissue transcriptomes between *Nme7+/−* and wild-type rats did not reveal, after correction for multiple testing, any other significantly differentially expressed genes. In the liver of *Nme7+/−* males, we observed 38 downregulated and 41 upregulated transcripts compared to wt (Supplementary Table S2). Apart from *Nme7*, hydroxysteroid (17-β) dehydrogenase 2 (*Hsd17b2*), cytochrome P450 7B1 (*Cyp7b1*, also known as oxysterol 7α-hydroxylase), and sulfotransferase 2A1 (*Sult2A1*) showed lower hepatic expression in *Nme7*+/– animals. Conversely, stearoyl-CoA desaturase 1 (*Scd1*), fatty acid desaturase 1 (*Fads1*), 7-dehydrocholesterol reductase (*Dhcr7*) or insulin-induced gene 1 (*Insig1*) were among the upregulated in *Nme7*+/– when compared to wild-type rats. The most pronounced differences in expression were corroborated by qPCR validation (Supplementary Figure S4). The most enriched disease and function classes were steroid metabolism and cholesterol metabolism (Benjamini–Hochberg corrected *p* = 4.28 × 10^−5^ and 1.85 × 10^−4^, respectively). In the analysis of potential upstream regulators (Supplementary Table S3), sterol regulatory element binding transcription factors 1 and 2 (*Srebf1, Srebf2*) stood out as the predicted major activated regulators (activation z-scores 3.09 and 2.23; *p* = 2.91 × 10^−11^ and *p* = 1.51 × 10^−10^, respectively) and cytochrome P450 oxidoreductase (*Por*) was predicted to be the most inhibited one (activation z-score −2.24; *p* = 5.22 × 10^−9^). Most of the above nodes and transcripts were recapitulated in the derived mechanistic network, reaching the highest consistency score (Figure 7).

## 4. Discussion

The *NME* gene family consists of ten members that have, originally, been implicated mainly in cancer and metastasis dissemination [21]. The evidence for their involvement in metabolic conditions has been scarce so far. A single nucleotide polymorphism (SNP) in *NME5* was associated with type 2 diabetes risk in a large, multiancestry meta-analysis [22]. *Nme1* was shown to regulate glucose-stimulated insulin secretion in an in vitro experiment [23]. Over ten distinct SNPs in *NME7* were found to be associated with blood pressure [24] and electrocardiographic traits [25], or venous thromboembolism [26,27] in several genome-wide association studies. We have previously demonstrated an association of *NME7* gene variants with insulin secretion and lipid spectrum changes in two independent Caucasian populations [8]. In a set of rat recombinant inbred models, the reduced expression of hepatic *Nme7* strongly correlated with metabolic disturbances and, on the network level, was connected to shifts of carbohydrate and lipid metabolism as well as ciliogenesis [10]. Functionally, Nme7 is found in ciliated structures and is a regulatory component of γ-tubulin ring complex [13], and this is reflected by the phenotypes observed in *Nme7-/-* mice [15,16] and rats [17], consistent with primary ciliary dyskinesia. Following up on our prior studies and given the observation that the *Nme7* knockout rats are not viable [17], we compared the metabolic profile of *Nme7+/*− rats to their wild-type controls in this study. The single-allele deficiency resulted in the reduced expression of *Nme7* in all tested tissues and metabolic and transcriptomic shifts in the heterozygous animals.

Morphologically, pancreatic islet fibrosis was the main finding in *Nme7+/*− male rats. Pancreatic islet fibrosis was first described in 40-week-old male Sprague–Dawley rats by Hajdu and Rona [28]. They speculated that the islet changes begin with an increased need for insulin followed by compensatory β-cell hyperplasia and islet enlargement, resulting in islet fibrosis no sooner than at the age of 40 weeks [29]. This seems like a plausible explanation for the early emergence of the phenotype in our model as well. The *Nme7+/*− male rats were gradually gaining more weight over time compared to their already heavy wild-type controls, and from the age of 9 weeks, this difference became statistically significant. The *Nme7+/*− male rats also became more glucose intolerant, as shown by the intraperitoneal glucose tolerance test at the age of 12 weeks. Altogether, this may have accelerated the development and manifestation of islet fibrosis present in more than 50% of male *Nme7+/*− rats at sacrifice. Even though the mechanism of age-related fibrosis in SD rat is still not fully elucidated, the α-SMA-positive myofibroblasts transformed from pancreatic stellate cells were shown to contribute to the development of fibrosis [30]. The stimuli contributing to the transformation of pancreatic stellate cells might be related to the unfavorable environment of obesity and glucose intolerance observed in *Nme7+/*− male rats as pancreatic islet fibrosis has also been observed in other rat diabetic models, including non-obese Goto–Kakizaki rats [31] or in mildly obese Otsuka Long–Evans Tokushima fatty rats [32]. Islet fibrosis with amyloid deposition was also described in humans [33].

Together with indices of impaired kidney function, we observed signs of tubular impairment in the heterozygous *Nme7+/*− animals. Damaged renal tubular epithelium undergoes a regeneration process where either remaining undamaged differentiated epithelial cells [34] or scattered tubular progenitor cells [35] renew the tubular epithelium. Differentiated epithelial cells de-differentiate and proliferate during repair and re-differentiate again when the process is complete. Using the transcription factors Oct4 and Sox2, the renal proximal tubular epithelial cells were successfully dedifferentiated into induced pluripotent stem cells [36]. Oct4 is one of the downstream targets of *Nme7*, the knockout of *Nme7* led to downregulation of Oct4 expression together with other transcription factors such as Nanog, Klf4, c-Myc, telomerase, Dnmt3B, Sox2, and Eras [37]. Therefore, one might speculate that the renal tubular epithelium defects found in *Nme7+/*− rats could be related to impaired tubule renewal due to downstream effects of reduced *Nme7* expression, even though the effects of the unfavorable obesity and glucose intolerance cannot be ruled out as well [38].

The observed combination of glucose tolerance defect and increased weight and adiposity in *Nme7+/−* rats was corroborated by substantial shifts of their hepatic transcriptome. While we did not detect any changes in serum lipid levels or signs of liver steatosis in these standard diet-fed animals, many of the dysregulated transcripts were related to lipid metabolism and its interface with insulin resistance. Therefore, the downregulation of *Cyp7b1* in *Nme7+/−* animals together with upregulation of lipogenic enzymes *Fads1, Dhcr7* and *Scd1* may reflect a switch towards increased lipid synthesis in the liver, corroborated by the predicted activation of sterol regulatory element-binding transcription factors 1 and 2. It was shown that the inability to upregulate CYP7B1 in the setting of insulin resistance results in the accumulation of toxic intracellular cholesterol metabolites that promote inflammation and hepatocyte injury [39]. Our observation is also consistent with the study showing *Fads1* knockout mice to be among the leanest of 3,651 chow-fed knockout lines analyzed for body composition and were among the most glucose tolerant of 2489 high-fat-diet-fed knockout lines analyzed by oral glucose tolerance test. *Fads1* knockout mice also showed lower fasting glucose, insulin, triglyceride, and total cholesterol levels [40]. The excess lipids were most likely preferentially stored in the adipose tissue, resulting in the increased adiposity and body weight of *Nme7+/−* heterozygotes. The mechanism of *Nme7* involvement is not clear, although the possible links were indicated by the outcomes of network analysis. *Nme7* interacts directly with several entities related to lipid handling and insulin sensitivity, including the established nodes of metabolic diseases: the mitogen-activated protein kinase kinase kinase kinase 4 (Mapk4k4) [41] and hepatocyte nuclear factor 4 (Hnf4) [42].

While the design of our current study did not allow us to elucidate the causal link between the *Nme7* variant and the observed changes on morphological, metabolic and gene expression levels, the results support, in concert with several previous observations, a potential implication of NME7 in pathogenesis of glucose intolerance and adiposity.

## Data Availability

The datasets generated during and/or analyzed during the current study are available from the corresponding author on reasonable request. The microarray data generated during and/or analyzed during the current study are available in the ArrayExpress repository (https://www.ebi.ac.uk/arrayexpress), Experiment ArrayExpress accession: E-MTAB-10011.

## Supplementary Materials

The following are available online at https://www.mdpi.com/article/10.3390/genes12071087/s1, Figure S1: The oral glucose tolerance test. Figure S2: Fasting serum concentrations of triacylglycerols, total cholesterol, HDL-cholesterol, alkaline phosphatase, aspartate aminotransferase, total protein, bilirubin, phosphate, calcium, iron and chloride in wild-type and *Nme7+/−* heterozygous male rats, Figure S3: Haematoxylin-eosin stained sections of liver and kidney in wild-type and *Nme7+/−* heterozygous adult male rats. Figure S4: qPCR validation of the microarray results. Table S1: Basic haematologic parameters of wild-type and *Nme7+/−* male rats. Table S2: Significantly differentially expressed genes in *Nme7+/−* vs. wild-type male rat livers. Table S3: Predicted activated or inhibited upstream regulators in *Nme7+/−* male rat livers.
