# Supplementary material for: Heterozygous Nme7 Mutation Affects Glucose Tolerance in Male Rats
Source: Genes (Basel). 2021 Jul 18;12(7):1087. doi: 10.3390/genes12071087 (PMC8305224; doi:10.3390/genes12071087)
Supplement: Supplementary file 1 [file genes-12-01087-s001.zip › genes-1288883-supplementary.pdf]

Supplementary Table S1:

Basic haematologic parameters of *Nme7+/–* and wild-type (wt) male rats

Trait	Male *Nme7+/-*	Male wt	P (t-test)
**WBC (10^9/L)**	10.8±1.5	6.2±0.3	0.042
**Neu (%)**	12.0±0.8	13.4±1.6	n.s.
**Lym (%)**	80.8±0.9	79.2±1.4	n.s.
**Mon (%)**	5.5±0.4	6.1±1.1	n.s.
**Eos (%)**	1.6±0.2	1.3±0.3	n.s.
**Bas (%)**	0.1±0.1	0	n.s.
**Neu#**	1.21±0.13	0.82±0.08	n.s.
**Lym#**	8.83±1.24	4.94±0.23	0.044
**Mon#**	0.58±0.08	0.40±0.08	n.s.
**Eos#**	0.18±0.04	0.08±0.02	n.s.
**Bas#**	0.03±0.02	0	n.s.
**RBC (10^{12}/L)**	8.04±0.14	8.27±0.27	n.s.
**HGB (g/L)**	144.5±2.6	146.4±3.3	n.s.
**HCT (%)**	44±1	45±1	n.s.
**MCV (fL)**	54.6±0.3	54.3±0.6	n.s.
**MCH (pg)**	18.0±0.1	17.8±0.2	n.s.
**MCHC (g/L)**	329±2	327±1	n.s.
**RDW-CW (%)**	0.123±0.002	0.118±0.002	n.s.
**RDW-SD (fL)**	27.9±0.5	26.7±0.5	n.s.
**PLT (10^3/μL)**	622±48	560±57	n.s.
**MPV (fL)**	5.67±0.09	5.86±0.16	n.s.

Supplementary Table S1. Basic haematologic parameters of *Nme7+/-* (n=10) and wild-type (wt, n=17) male rats. Data are expressed as mean ± S.E.M. The significance levels of unpaired (two-tailed) Student t test are indicated in the third column, n.s.: not significant. WBC: white blood cells; Neu (%): neutrophil percent; Lym (%): lymphocyte percent; Mon (%): monocyte percent; Eos (%): eosinophil percent; Bas (%): basophil percent; Neu#: neutrophil number; Lym#: lymphocyte number; Mon#: monocyte number; Eos#**:** eosinophil number; Bas#**:** basophil number; RBC: red blood cells; HGB: hemoglobin; HCT: hematocrit; MCV: mean cell volume; MCH: mean cell hemoglobin; MCHC: mean cell hemoglobin concentration; RDW-CW: Red blood cell distribution width **-** coefficient of variation; RDW-SD: Red blood cell distribution width – standard ; PLT: platelet count; MPV: mean platelet volume.

Supplementary Table S2. Significantly differentially expressed genes in *Nme7+/-* vs. wildtype male rat livers.

Symbol	Entrez Gene Name	Affymetrix ID	FDR p-value	Fold Change (*Nme7+/-* vs. wildtype)
SULT2A1	sulfotransferase family 2A member 1	17629978	0.0447	-9.451
DBP	D-box binding PAR bZIP transcription factor	17616470	0.0102	-6.38
CYP7B1	cytochrome P450 family 7 subfamily B member 1	17746437	0.00133	-4.323
SCPEP1	serine carboxypeptidase 1	17659910	0.000127	-2.846
HSD17B2	hydroxysteroid 17-beta dehydrogenase 2	17733868	0.0258	-2.554
LOC102549726	uncharacterized LOC102549726	17752880	0.00901	-2.304
POLR2H	RNA polymerase II subunit H	17670333	0.0222	-2.155
Gm21596/Hmgb1	high mobility group box 1	17734560	0.0357	-2.12
AKR1C4	aldo-keto reductase family 1 member C4	17720497	0.0193	-2.106
UGT2B11	UDP glucuronosyltransferase family 2 member B11	17689096	0.0396	-1.991
TOM1L1	target of myb1 like 1 membrane trafficking protein	17660006	0.0119	-1.915
Cyp3a73	cytochrome P450, family 3, subfamily a, polypeptide 73	17679219	0.0154	-1.806
GAS5	growth arrest specific 5	17681329	0.000238	-1.803
RGD1563620	similar to retinoblastoma binding protein 4	17739166	0.00133	-1.747
CA14	carbonic anhydrase 14	17749573	0.0308	-1.744
FMO5	flavin containing dimethylaniline monoxygenase 5	17741150	0.0252	-1.727
MT-ND6	NADH dehydrogenase, subunit 6 (complex I)	17867334	0.0434	-1.727
C5orf63	chromosome 5 open reading frame 63	17726352	0.0356	-1.717
Ctdspl	CTD small phosphatase like	17848680	0.00711	-1.65
Plekha6	pleckstrin homology domain containing A6	17680180	0.00179	-1.624
MRPL55	mitochondrial ribosomal protein L55	17646298	0.00773	-1.6
LOC108351703	60S ribosomal protein L27a-like	17852354	0.000162	-1.596
Teddm2	transmembrane epididymal family member 2	17685367	0.0197	-1.596
mir-568	microRNA 568	17669026	0.012	-1.593
LGR4	leucine rich repeat containing G protein-coupled receptor 4	17764547	0.00766	-1.592
Obp3	alpha-2u globulin PGCL4	17807836	0.0431	-1.582
SDS	serine dehydratase	17678273	0.0237	-1.568
ATP11C	ATPase phospholipid transporting 11C	17879544	0.00628	-1.554
MINPP1	multiple inositol-polyphosphate phosphatase 1	17629911	0.0274	-1.548
SNRPB2	small nuclear ribonucleoprotein polypeptide B2	17766763	0.0213	-1.548
EIF2S1	eukaryotic translation initiation factor 2 subunit alpha	17816934	0.0304	-1.544
NME7	NME/NM23 family member 7	17681568	0.0195	-1.535
ELOC	elongin C	17805244	0.0244	-1.529
DYNLL1	dynein light chain LC8-type 1	17674442	0.00682	-1.511
ASF1A	anti-silencing function 1A histone chaperone	17755165	0.0132	-1.505
H4C4	H4 clustered histone 4	17795957	0.00412	-1.504
THOC7	THO complex 7	17697504	0.0176	-1.504
Tmem258/Tmem258b	transmembrane protein 258	17624009	0.0000783	-1.504

Symbol	Entrez Gene Name	Affymetrix	FDR p-value	Fold Change (*Nme7+/-* vs. wildtype)
SECTM1	secreted and transmembrane 1	17664120	0.0101	1.501
AIFM2	apoptosis inducing factor mitochondria associated 2	17755045	0.0241	1.505
TUBB2A	tubulin beta 2A class IIa	17715339	0.0121	1.523
PLVAP	plasmalemma vesicle associated protein	17710743	0.0106	1.535
CRYBG1	crystallin beta-gamma domain containing 1	17759371	0.000792	1.539
HMOX1	heme oxygenase 1	17728071	0.0132	1.541
LOC100361180	40S ribosomal protein S17-like	17704784	0.00453	1.555
PGRMC2	progesterone receptor membrane component 2	17747086	0.0199	1.555
GAS6	growth arrest specific 6	17709230	0.0151	1.558
CORO1C	coronin 1C	17674643	0.0341	1.579
ZNF385A	zinc finger protein 385A	17841635	0.0387	1.584
COPS9	COP9 signalosome subunit 9	17866539	0.00034	1.599
Chtopl1	chromatin target of PRMT1-like 1	17796579	0.0125	1.599
IGF2BP3	insulin like growth factor 2 mRNA binding protein 3	17791224	0.0321	1.609
SLC13A3	solute carrier family 13 member 3	17779409	0.0186	1.616
LCN1	lipocalin 1	17770733	0.000423	1.619
MAPKAPK2	MAPK activated protein kinase 2	17684022	0.00224	1.623
RORA	RAR related orphan receptor A	17845526	0.022	1.628
CSRP1	cysteine and glycine rich protein 1	17680410	0.0363	1.642
IL18BP	interleukin 18 binding protein	17635431	0.0329	1.642
RBM34	RNA binding motif protein 34	17734548	0.0000572	1.646
mir-328	microRNA 328	17733040	0.0212	1.648
mir-126	microRNA 126	17770717	0.00524	1.651
RPS24	ribosomal protein S24	17705621	0.0112	1.661
CA7	carbonic anhydrase 7	17731315	0.0196	1.665
RNF125	ring finger protein 125	17721797	0.0308	1.711
NPAS2	neuronal PAS domain protein 2	17858614	0.0217	1.813
CA1	carbonic anhydrase 1	17737075	0.00262	1.859
FDPS	farnesyl diphosphate synthase	17748801	0.0446	1.872
FADS1	fatty acid desaturase 1	17623991	0.0443	1.899
INSIG1	insulin induced gene 1	17788437	0.0284	1.970
CYP2J2	cytochrome P450 family 2 subfamily J member 2	17808722	0.000325	2.007
HMGCS1	3-hydroxy-3-methylglutaryl-CoA synthase 1	17735993	0.0259	2.172
CDKN1A	cyclin dependent kinase inhibitor 1A	17753672	0.0236	2.172
DHCR7	7-dehydrocholesterol reductase	17622570	0.0152	2.493
ZNF442	zinc finger protein 442	17688200	0.0396	2.504
SLC13A5	solute carrier family 13 member 5	17658370	0.0242	2.574
MGC108823	similar to interferon-inducible GTPase	17723515	0.045	2.662
OVOS2	alpha-2-macroglobulin like 1 pseudogene	17795265	0.00699	3.315
CYP2B6	cytochrome P450 family 2 subfamily B member 6	17614499	0.0377	4.590
SCD1	stearoyl-CoA desaturase	17642329	0.0452	5.164

Supplementary Table S3. Predicted activated or inhibited upstream regulators in *Nme7+/-* male rat livers.

Upstream Regulator	Molecule Type	Predicted Activation State	Activation z-score	p-value of overlap	Target Molecules in Dataset
POR	enzyme	Inhibited	-2.236	5.22E-09	CYP2B6,CYP7B1,DHCR7,FDPS,HMGCS1,HMOX1,INSIG1,SCD,SDS
ADRB	group	Inhibited	-2.219	8.38E-05	CDKN1A,FDPS,HMGCS1,HMOX1,INSIG1
PROM1	other	Inhibited	-2.000	1.17E-07	DHCR7,FDPS,HMGCS1,INSIG1
MFSD2A	transporter		-1.980	6.18E-07	DHCR7,FADS1,INSIG1,SCD
CFTR	ion channel		-0.555	6.64E-06	CYP2B6,FADS1,FDPS,HSD17B2,SCD
OGA	enzyme		-0.538	5.05E-05	AIFM2,CDKN1A,CYP2B6,DHCR7,FDPS,GAS6,HMGCS1,IGF2BP3
NR1I3	ligand-dependent nuclear receptor		0.077	1.19E-06	CDKN1A,CYP2B6,CYP7B1,INSIG1,SCD,SULT2A1
RXRA	ligand-dependent nuclear receptor		0.195	4.51E-06	CDKN1A,CYP2B6,GAS6,HMOX1,INSIG1,RORA,SCD,SULT2A1
PPARA	ligand-dependent nuclear receptor		0.954	1.94E-08	CDKN1A,CYP2B6,CYP7B1,DHCR7,DYNLL1,FADS1,FDPS,HMGCS1,HMOX1,INSIG1
PPARGC1A	transcription regulator		1.526	2.06E-05	CDKN1A,CYP2B6,FDPS,GAS6,HMOX1,INSIG1,SCD,SULT2A1
MAP2K5	kinase		1.982	2.13E-05	DHCR7,FDPS,HMGCS1,INSIG1
NR1I2	ligand-dependent nuclear receptor	Activated	2.024	2.19E-09	CDKN1A,CYP2B6,DHCR7,FMO5,HMGCS1,INSIG1,PGRMC2,SCD,SULT2A1
CEBPA	transcription regulator	Activated	2.030	1.50E-06	CDKN1A,CYP2B6,DHCR7,HMOX1,MAPKAPK2,RORA,SCD,SECTM1,SULT2A1,TUBB2A
SCAP	other	Activated	2.183	2.73E-07	CYP2B6,DHCR7,FDPS,HMGCS1,INSIG1,SCD
MAPK7	kinase	Activated	2.219	4.61E-06	DHCR7,FDPS,HMGCS1,HMOX1,INSIG1
SREBF2	transcription regulator	Activated	2.626	5.27E-09	CDKN1A,CYP7B1,DHCR7,FDPS,HMGCS1,INSIG1,SCD
SREBF1	transcription regulator	Activated	2.931	4.46E-10	CDKN1A,CSRP1,CYP7B1,DHCR7,FADS1,FDPS,HMGCS1,HMOX1,INSIG1,RPS24

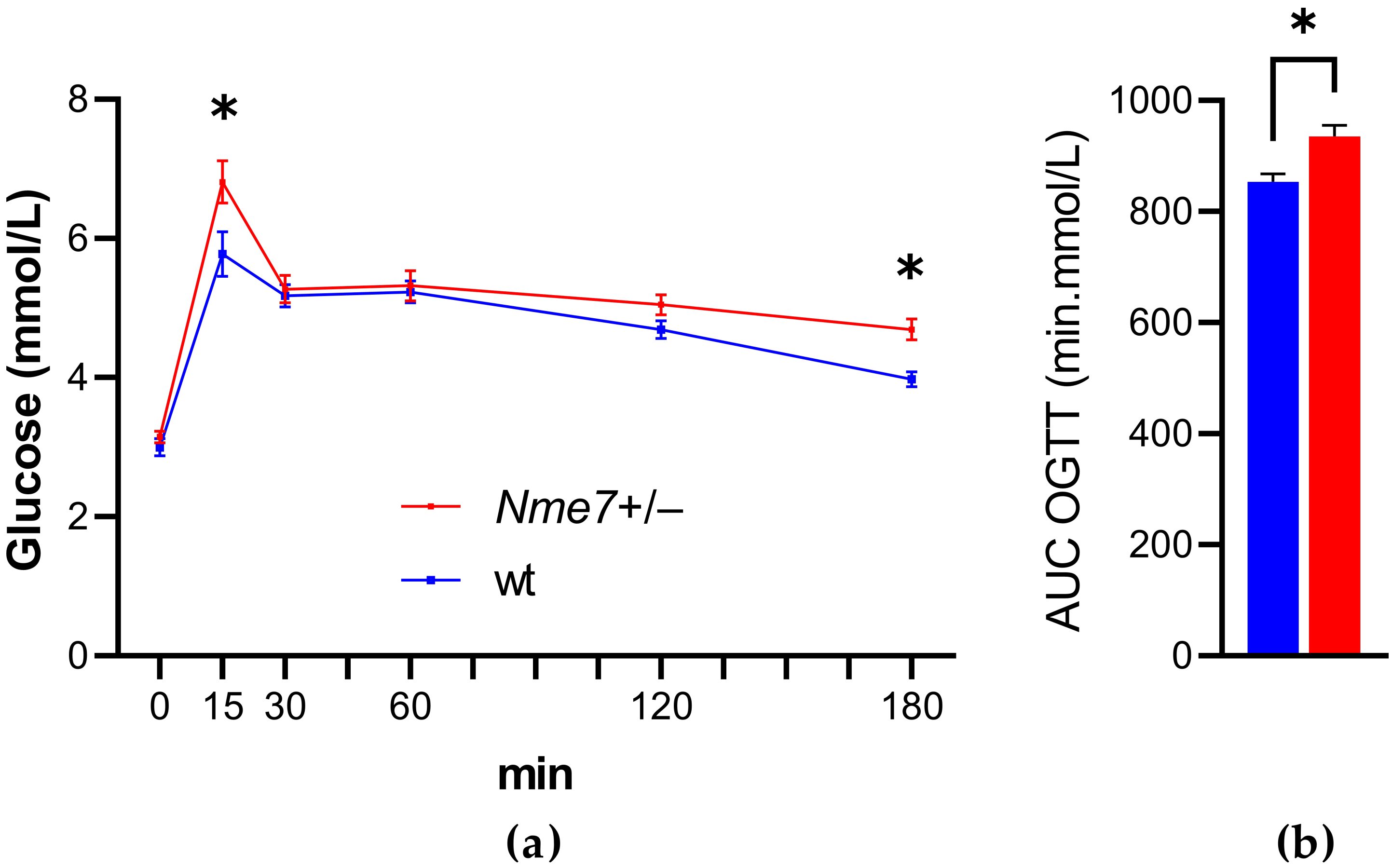

Supplementary Figure S1. The oral glucose tolerance test (OGTT). (a) The course of glycaemic curves in wild-type (wt, blue, n=10) and *Nme7+/–* heterozygous (*Nme7+/–*, red, n=17) male) rats. (b) Areas under the glycaemic curves (AUC) in wild-type (wt, blue bars) and *Nme7+/–* heterozygous (*Nme7+/–*, red bars) rats. Data are expressed as mean ± SEM; the significance levels for strain comparison using the repeated measures ANOVA (OGTT) or the unpaired (two-tailed) Student t test (AUC, insulin) are indicated as follows: * $p < 0.05$.

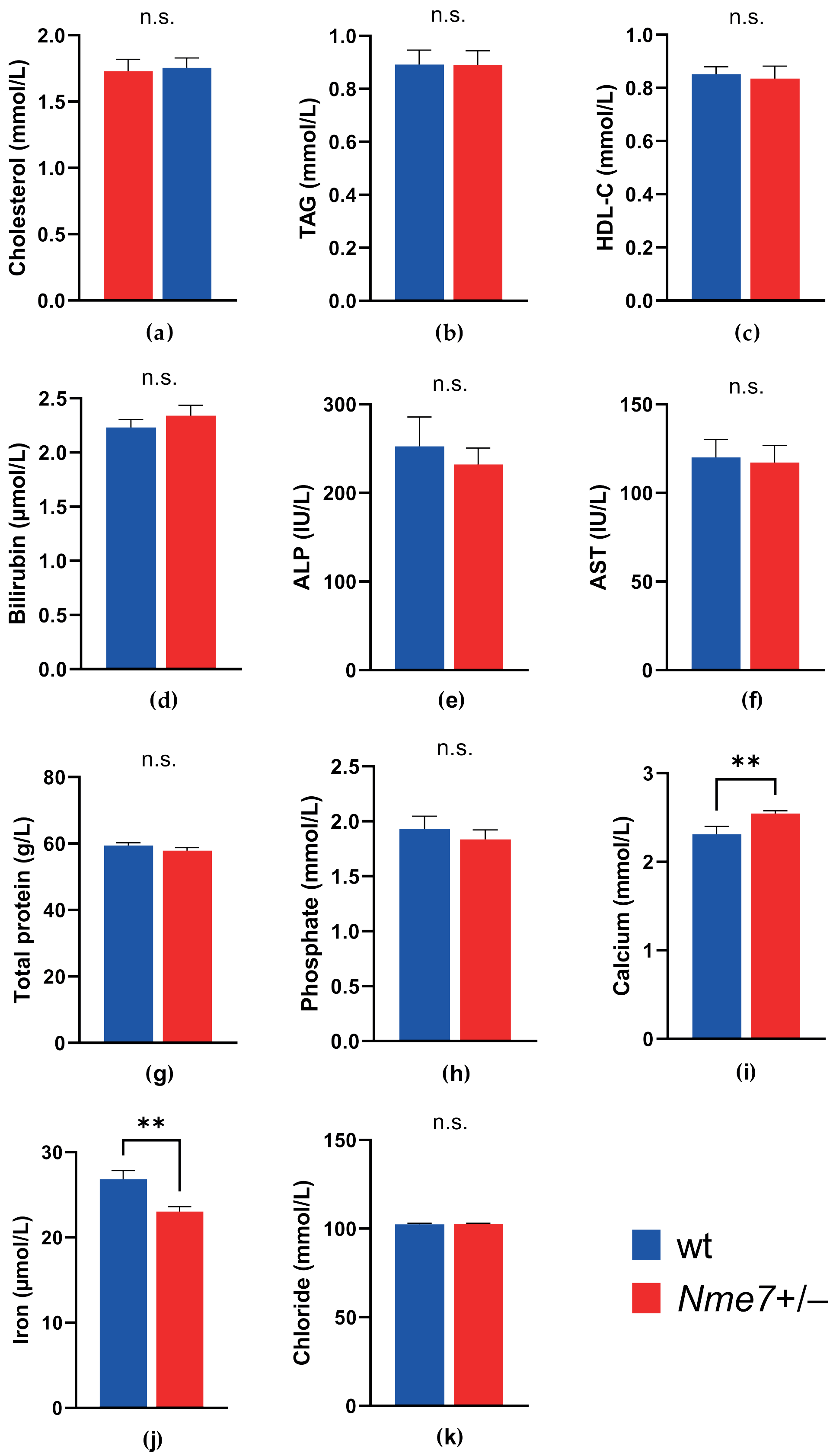

Supplementary Figure S2. Fasting serum (a) cholesterol; (b) triacylglycerols (TAG); (c) high-density lipoprotein cholesterol (HDL-C); (d) bilirubin ; (e) alkaline phosphatase (ALP); (f) aspartate aminotransferase (AST); (g) total protein; (h) phosphate; (i) calcium; (j) iron; (k) chloride in wild-type (wt, blue, n=10) and *Nme7+/–* heterozygous (*Nme7+/–*, red, n=17) male rats. Data are expressed as mean ± SEM; the significance levels for strain compa-rison using the unpaired (two-tailed) Student t test are indicated as [illegible]

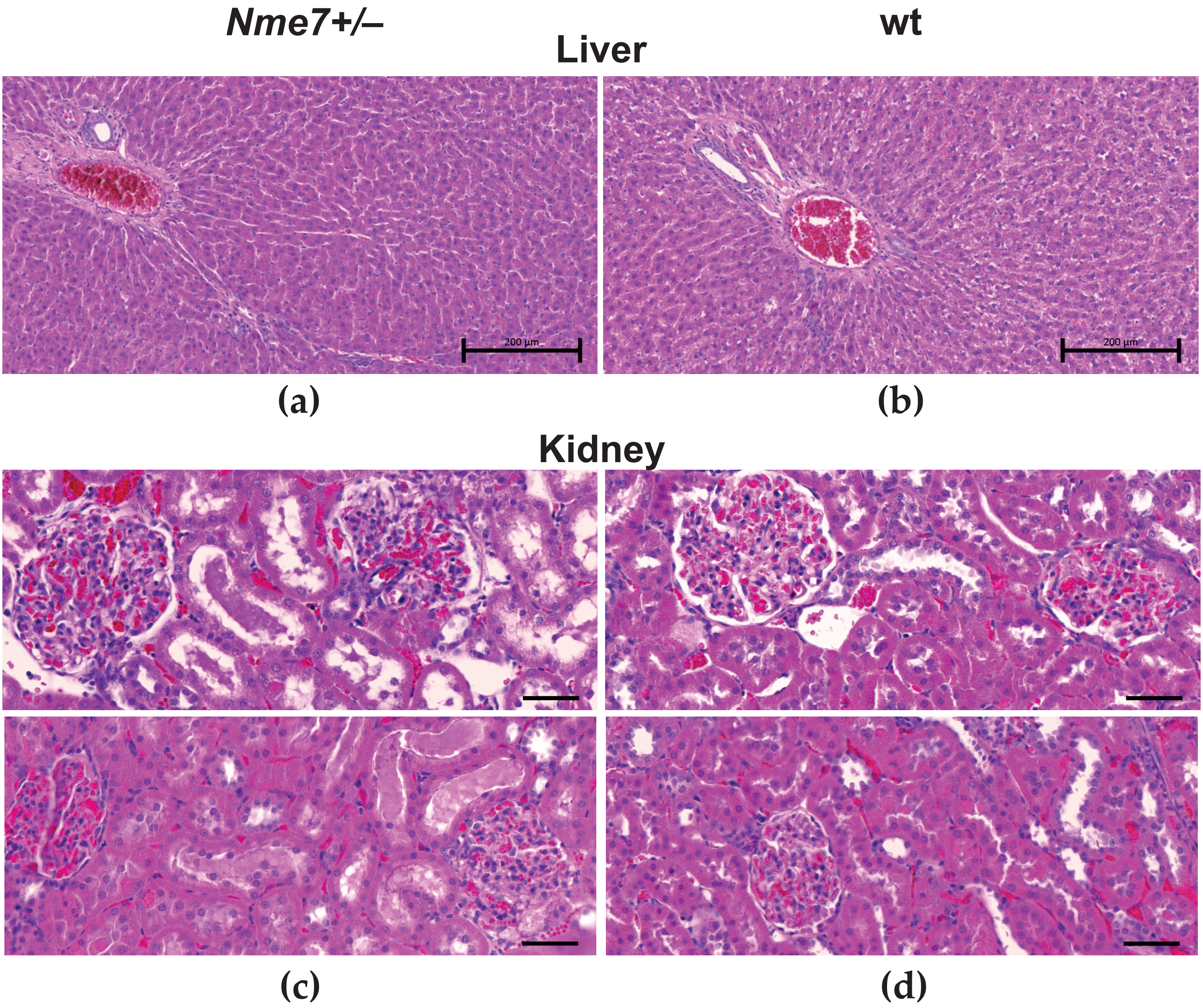

Supplementary Figure 3. Haematoxylin-eosin stained sections of (a,b) liver; (c,d) kidney in *Nme7+/–* heterozygous (*Nme7+/–*, a,c) and wild-type (**wt,** b,d) adult male rats.

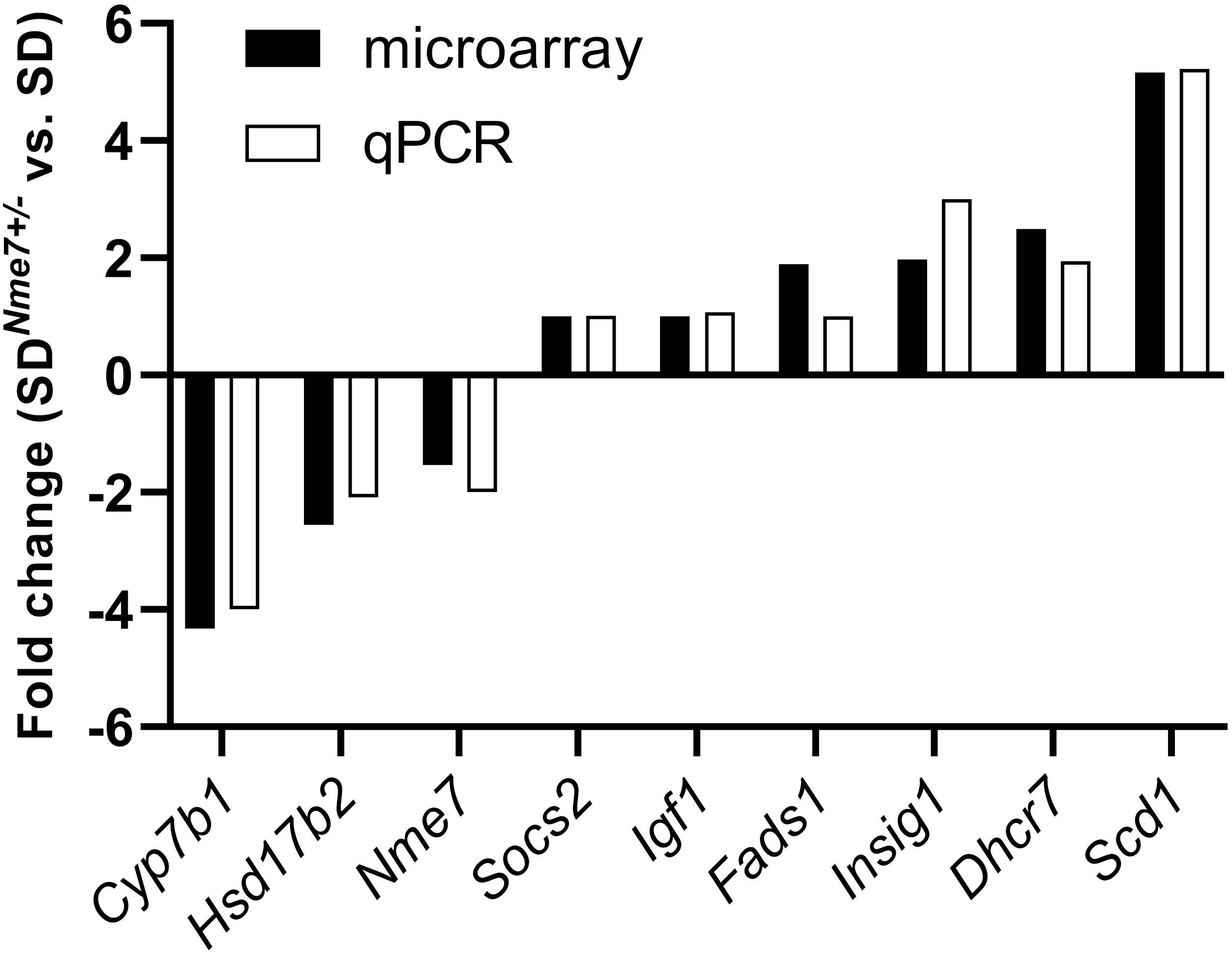

Supplementary Figure S4: Validation of transcriptomic results by qPCR. Fold changes are indicated for comparison of expression between $SD^{Nme7-/-}$ vs. SD male rats (qPCR: open bars; microarray: full bars). The microarray fold-change values are shown based on Partek Genomics Suite analysis, the qPCR results are expressed as the mean fold-change for each transcript (see Methods). The gene symbols are used in accordance with the names approved by the HUGO Gene Nomenclature Committee: cytochrome P450, family 7, subfamily b, polypeptide 1 (*Cyp7b1*), hydroxysteroid 17-beta dehydrogenase 2 (*Hsd17b2*), NME/NM23 family member 7 (*Nme7*), suppressor of cytokine signaling 2 (*Socs2*), insulin-like growth factor 1 (*Igf1*), fatty acid desaturase (*Fads1*), insulin induced gene 1 (*Insig1*), 7-dehydrocholesterol reductase (*Dhcr7*), stearoyl-Coenzyme A desaturase 1 (*Scd1*).
